# Exposure to a PFOA, PFOS and PFHxS Mixture during Gestation and Lactation Alters the Liver Proteome in Offspring of CD-1 Mice

**DOI:** 10.3390/toxics12050348

**Published:** 2024-05-09

**Authors:** Emily Kaye, Emily Marques, Juliana Agudelo Areiza, Seyed Mohamad Sadegh Modaresi, Angela Slitt

**Affiliations:** 1 Department of Biomedical and Pharmaceutical Sciences, College of Pharmacy, University of Rhode Island, 7 Greenhouse Rd, Kingston, RI 02881, USA; emilykaye@uri.edu (E.K.); 21saraemma@gmail.com (E.M.); juliana_agudelo@uri.edu (J.A.A.); smodaresi@uri.edu (S.M.S.M.); 2 Office of Pollution Prevention and Toxics, US EPA, 1200 Pennsylvania Ave. NW, Washington, DC 20460, USA

**Keywords:** perfluoroalkyl substance, PFAS, peroxisome proliferator, non-alcoholic fatty liver disease, perinatal, liver injury

## Abstract

Perfluroalkyl substances (PFASs) are persistent man-made chemicals considered to be emerging pollutants, with Perfluorooctanoic acid (PFOA), Perfluorooctanesulfonic acid (PFOS), and Perfluorohexanesulphonic acid (PFHxS) being linked to hepatotoxicity and steatosis. PFOA, PFOS, and PFHxS can undergo placental and lactational transfer, which results in PFOA, PFOS, and PFHxS distribution to the neonatal liver. Moreover, in pregnant dams, exposure to a PFAS mixture, in combination with a high fat diet, increased hepatic steatosis in offspring at postnatal day 21, but the mechanisms have not been elucidated. It was hypothesized that gestational/lactational PFAS exposure would alter the pup liver proteome and biochemical/signaling pathways. Timed-pregnant CD-1 dams were fed a standard chow or 60% kcal high-fat diet. From GD1 until PND20, dams were dosed via oral gavage with vehicle (0.5% Tween 20), individual doses of PFOA, PFOS, PFHxS at 1 mg/kg, or a mixture (1 mg/kg each, totaling 3 mg/kg). Livers were collected from PND21 offspring and SWATH-MS proteomics was performed. IPA analysis revealed PFAS exposure modified disease and biological function pathways involved in liver damage, xenobiotics, and lipid regulation in the PND21 liver. These pathways included lipid and fatty acid transport, storage, oxidation, and synthesis, as well as xenobiotic metabolism and transport, and liver damage and inflammation. This indicates the pup liver proteome is altered via maternal exposure and predisposes the pup to metabolic dysfunctions.

## 1. Introduction

Perfluoroalkyl Substances (PFAS) are persistent man-made environmental pollutants that are present in aqueous film-forming foams (AFFF); non-stick pots, pans and utensils; water and stain-resistant clothing; carpeting and carpet treatments; takeout food containers; and cosmetics. They are considered to be ubiquitously present in the environment [1]. An overarching health concern for PFAS is bioaccumulation, with Perfluorooctanoic Acid (PFOA), Perfluorooctanesulfonic Acid (PFOS) and Perfluorohexanesulfonic Acid (PFHxS) possessing half-lives in humans that range from 1.3–8.5 years [2,3,4]. Among the various PFAS, PFOA, PFOS, and PFHxS are the most frequently detected and abundant in human serum samples [5]. Multiple animal and human studies report adverse health outcomes associated with PFOA and PFOS exposure, such as liver injury, decreased immunity, dyslipidemia, obesity, and increased risks of testicular and kidney cancer; PFHxS is also associated with liver injury and immunotoxicity [1,6,7,8,9,10].

PFOA, PFOS, and PFHxS accumulate in the liver, and there are established links between PFAS serum levels and elevated serum liver injury markers in humans and pre-clinical animal models [7,9,11,12,13,14]. Additionally, all three can induce hepatocyte peroxisome proliferation, liver hypertrophy, vacuolization, and hyperplasia in rats and mice [9,15]. Both PFOA and PFOS are associated with serum ALT in humans, indicating an association with liver injury/cytotoxicity in humans [16,17]. Recent work utilizing the Emory University Pediatric Liver Biopsy Data Repository (2007–2015) examined liver biopsies from 74 children (7–19 years-of-age) clinically diagnosed with non-alcoholic fatty liver disease (NAFLD) and quantified serum PFOA, PFOS, and PFHxS. The authors concluded that PFAS exposure predominantly dysregulated multiple lipid and amino acid pathways that are associated with NAFLD pathogenesis, with serum PFOS and PFHxS being associated with increased odds of having non-alcoholic steatohepatitis (NASH) [18]. PFOA and PFOS have been shown to activate multiple nuclear receptors (i.e., Peroxisome proliferator-activated receptor alpha (PPARA), Constitutive androstane receptor (CAR), Pregnane X receptor (PXR), Liver X receptor α (LXRα), and Farnesoid X receptor (FXR)), and metabolic pathways in rodent and primary human hepatocytes [19]. PFOS and PFHxS administration have been associated with hepatic transcriptome and proteome alterations [20,21]; previous work in adult male C57BL/6 mice has demonstrated modulation of fatty acid beta oxidation, lipid metabolism, and xenobiotic metabolism pathways at the proteomic level [22].

The “Developmental Origins of Health and Disease” concept emphasizes the significance of pollutant exposures throughout fetal development in provoking metabolic alterations and increased disease risk, even after the exposure has occurred [23]. PFOA, PFOS, and PFHxS can transverse the placental membrane and induce developmental toxicity in animal models and humans [24,25,26], and have also been detected in human breast milk [27]. PFOA, PFOS and PFHxS exposure has also been associated with altered placental function and decreased birth weights [28]. Whereas PFOA, PFOS, and PFHxS effects are well described in adult mice, few studies have examined how exposure during development subsequently impacts liver function in offspring. PFOA has been shown to induce hepatocellular hypertrophy and liver lesions in CD-1 pups [29]. Gestational and lactational PFOS exposure increased liver weights in male and female pups at postnatal day (PND) 21. These changes were associated with increased liver mRNA expression of Cyp4a14, lipoprotein lipase (Lpl) and fatty acid translocase (Cd36) [12]. Moreover, gestational and lactational PFHxS exposure upregulated transcripts associated with lipid and xenobiotic metabolism (i.e., Acox1, Ehhadh, CD36, ApoA1, Cyp2b10, Sult1d1, Srebf1, and Ugt1a1) in livers of CD-1 offspring at PND36 [30]. Work from our group has shown that maternal exposure to a PFAS mix and high fat diet increased lipid content in livers of offspring at PND21 [31], and the livers from the latter study have been further characterized herein. While it has been observed that developmental exposure to PFOS or PFHxS can modulate the transcriptome, there are no published studies that have addressed whether PFAS administration modulates the liver proteome in neonates.

In the present study, timed-pregnant CD-1 dams were administered PFOA, PFOS and/or PFHxS, whether individually or as a mixture, from gestational day (GD) 1 through PND 21, as previously described [32]. The aims of the study herein are to (1) assess the influence of maternal PFAS exposure (during gestation and lactation) on the neonatal liver proteome; (2) reveal pathways modulated by individual PFAS and/or a PFAS mixture; (3) investigate the additive, antagonistic, and/or synergistic effects of the PFAS mixture; and (4) examine if maternal high fat diet influences the response of the neonatal liver to PFAS.

## 2. Materials and Methods

Chemicals. The PFAS chemicals used in the dosing solutions were purchased from Sigma Aldrich (St. Louis, MO, USA): PFOS, (Heptadecafluorooctanesulfonic acid potassium salt, CAS# 2795-39-3, Catalog# 89374, ≥98.0% purity, ~70% linear and ~30% branched isomers based on LC-MS/MS analysis, PFOA (Perfluorooctanoic acid, CAS# 335-67-1, Catalog# 171468, 95% purity), and PFHxS (Tridecafluorohexane-1-sulfonic acid potassium salt, CAS# 3871-99-6, Catalog# 50929, ≥98.0% purity). Iodoacetamide [IAA], Sodium Deoxycholate [NaDOC], Dithiothreitol [DTT], Formic Acid, Chloroform, Methanol, Urea, and Ammonium Bicarbonate were also purchased from Sigma Aldrich (St. Louis, MO, USA). Trypsin (TPCK-treated trypsin) was purchased from Sciex (Framingham, MA, USA).

**Figure 1:**
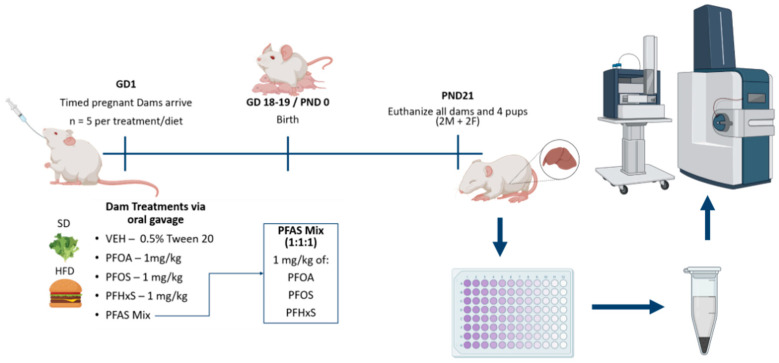
Treatment Paradigm. Timed pregnant CD-1 dams were administered either VEH, PFOA (1 mg/kg), PFOS (1 mg/kg), PFHxS (1 mg/kg), or a PFAS Mix (1 mg/kg PFOA, PFOS, and PFHxS) and then assigned to standard rodent diet (SD) or 60% kCal high fat diet chow (HFD). Protein was then isolated and digested from liver tissue, followed by mass spectrometry for proteome measurement. Treatments included: VEH SD (*n* = 10), PFOA SD (*n* = 10), PFOS SD (*n* = 8), PFHxS SD (*n* = 8), PFAS mix SD (*n* = 6), VEH HFD (*n* = 10), PFOA HFD (*n* = 12), PFOS HFD (*n* = 6), PFHxS HFD (*n* = 10), PFAS mix HFD (*n* = 8). Figure was made with BioRender (https://www.biorender.com/, accessed on 27 April 2024).

Treatment paradigm. Banked CD-1 mouse liver tissues from PND21 offspring from a previously described study were used for the study herein [31]. All animal protocols were approved by the University of Rhode Island’s (URI) Institutional Animal Care and Use Committee (IACUC). Administration of 1 mg/kg/day was selected based on previous work that demonstrated altered postnatal growth and development in CD-1 pups [32]. Administration of 1 mg/kg/day is also modestly higher than the lowest observed adverse effect level (LOAEL) described to induce liver enlargement in dams [12]. The mice were housed in a temperature-controlled room (20–26 °C) with relative humidity (30–70%) and lighting (12 h, light-dark cycles). Timed pregnant CD-1 dams (weighing 25–30 g) sourced from Charles River Laboratories (Wilmington, MA, USA) were administered either 0.5% Tween 20 vehicle (VEH), PFOA, PFOS, PFHxS, or a PFAS mixture daily through PND20. PFOA, PFOS, and PFHxS were individually prepared in VEH at a concentration of 1 mg/mL. A mixture of PFOA, PFOS and PFHxS was used to make a 3 mg/kg PFAS mixture at a 1:1:1 ratio of 1 mg/kg each individual compound. On PND21, all dams, two male, and two female pups were euthanized via cardiac puncture followed by cervical dislocation (Figure 1). Liver sections were snap frozen in liquid nitrogen and stored at −80 °C until processing and analysis. To summarize, the treatment groups were as follows:

(1) Standard rodent diet (SD) + vehicle (VEH, 0.5% Tween 20, 10 mL/kg), *n* = 10
(2) SD + PFOA (1 mg/kg), *n* = 10
(3) SD + PFOS (1 mg/kg), *n* = 8
(4) SD + PFHxS (1 mg/kg) *n* = 8
(5) SD + PFAS mix (1 mg/kg PFOA, PFOS, and PFHxS), *n* = 6
(6) 60% kCal high fat diet chow (HFD) + VEH, *n* = 10
(7) HFD + PFOA (1 mg/kg), *n* = 12
(8) HFD + PFOS (1 mg/kg), *n* = 6
(9) HFD + PFHxS (1 mg/kg), *n* = 10
(10) HFD + PFAS mix (1 mg/kg PFOA, PFOS, and PFHxS), *n* = 8

Bicinchoninic Acid (BCA) Assay. Protein concentration was determined by a BCA assay. The Pierce BCA Protein Assay Kit (Catalog# 23225) was purchased from ThermoFisher Scientific. Protein samples were isolated from the snap frozen pup livers, cut into ~25 mg sections and homogenized in 500 µL of 8M urea buffer (10 M Urea, 1 M triethylammonium bicarbonate [TEAB], qs to final volume with MilliQ-purified water) using the OMNI International (Kennesaw, GA, USA) Bead Ruptor Elite bead mill homogenizer at 5 m/s for 30 s. The BCA assay was performed according to the manufacturer’s instructions. The protein lysates were then diluted to 2 mg/mL of protein 8 M urea.

Proteomics Sample Preparation. After protein concentration was determined by BCA assay, the homogenates were diluted to a concentration of 2 mg/mL protein/sample for a starting volume of 100 µL. Then, each sample was spiked with 10 µL of 0.2 mg/mL bovine serum albumin (BSA) and denatured with the addition of 25 µL of 100 mM dithiothreitol (DTT) in a shaking water bath (100 rpm) for 15 min. Following denaturation, the samples were then incubated in the dark for 30 min with 25 µL of iodoacetamide (IAA) to reduce cysteine residues to the sulfhydryl form. Next, 250 µL of MilliQ-purified water and 500 µL of ice-cold methanol were added to each sample, followed by 250 µL of ice-cold chloroform to precipitate out protein and remove lipids and nucleic acids. The samples were centrifuged at 12,000 rpm for 5 min at 10 °C to create a phase separation. Once finished, a protein pellet in between the aqueous and organic layers was formed. All liquid was removed and the pellet was subsequently washed very gently with 500 µL ice-cold methanol and briefly dried before adding in 130 µL of sodium deoxycholate (NaDOC) in 50 mM ammonium bicarbonate (3% *w*/*v* solution) to lyse the proteins. A tube of lyophilized trypsin was then resuspended with 500 µL of MilliQ-purified water. From there, 12.5 µL of lyophilized trypsin was added to each sample, vortexed, and 138 µL of the sample was added to a MT-96 PCT MicroTubesTM (Pressure BioScience Inc, South Easton, MA, USA) before being placed in the Barocycler NEP2320 PCT Sample Preparation System (Source Scientific, Irvine, CA, USA). The following barocycler program was used: Time 1 = 50 s, Time 2 = 10 s, Pressure = 35 psi, Temp = 33 °C, Cycles = 75 (run one) and 60 (run two). A Haake SC 100 (ThermoFisher Scientific, Waltham, MA, USA) water bath was utilized to bring the water up to temp for use in the barocycler. The samples were then subjected to the barocycler for run one, then 12.5 µL of lyophilized trypsin was added before samples were subjected to the barocycler for a second run. Then, 135 µL of sample and 15 µL of 5% formic acid were added to a microcentrifuge tube, vortexed, and centrifuged at 12,000 rpm for 10 min at 10 °C. After centrifugation, 75–100 µL of the supernatant was very carefully removed and transferred into HPLC vials.

SWATH LC/MS. SWATH-DIA proteomics was conducted, as previously published [33,34]. A SCIEX 5600 TripleTOF mass spectrometer in positive electrospray ionization mode equipped with a DuoSpray ion source (SCIEX, Concord, ON, Canada) coupled to Acquity UHPLC HClass system (Waters Corp., Milford, MA, USA) was used to acquire pup liver sample data. SWATH–MS allows for retrospective mining of data by creating a digital repository of fragmented precursors within a predefined *m*/*z*. A mouse reference spectral library is then used for comparison against the generated dataset and Spectronaut^TM^ version 18 software transforms the raw data output into tangible protein intensities. The peptides were differentiated on an Acquity UHPLC Peptide BEH C18 (2.1 × 150 mm^2^, 300 Å, 1.7 μm) equipped with Acquity VanGuard precolumn (2.1 × 5 mm^2^, 300 Å, 1.7 μm), with the analytical column temperature kept at 40 °C. Chromatographic separation was achieved with a linear gradient method runtime of 60 min at 100 μL/min. Mobile phase A was composed of 98% MilliQ-purified water, 2% acetonitrile, 0.1% formic acid, and mobile phase B contained 98% acetonitrile, 2% MilliQ-purified water, and 0.1% formic acid. The method ran as follows: a total of 0 to 3 min (98% A), 4–48 min (60% to 90% A), 49–52 min (20% A), from 53–60 min (98% A). The last 7 min of the method at 98% A allowed for the column to equilibrate before the start of the next run. Samples were kept at 10 °C in the autosampler and 10 μg of protein was loaded onto the column per injection. In between each batch of samples (~47 samples/batch), trypsin-digested β-galactosidase peptides were injected (∼30 pmol/injection) to monitor mass calibration of the TOF detector and normalization of intensity during relative quantification.

Post-acquisition data processing and statistical analysis. Spectronaut^TM^ (Biognosys, 8952 Schlieren, Switzerland) was utilized to process the resulting data files from the SWATH-MS runs. The raw files from Analyst (Sciex, Framingham, MA, USA) were converted into an HTRMS file format utilizing HTRMS Convert (Biognosys, 8952 Schlieren, Switzerland) in Spectronaut^TM^. A reference spectral library from the UP000000589_mice reference protein database was generated and then compared against samples. The DIA analysis was performed with the factory settings set to default, with minor changes—trypsin/P was selected for the peptide’s enzyme/cleavage rules, single hit proteins were excluded, and mus musculus (MGI) was specified for gene annotation. The resulting file is then exported for analysis.

The exported file from Spectronaut^TM^ was processed in Microsoft Excel 365. The raw intensities were converted to pmol/mg protein utilizing the total protein approach (TPA), as previously published [35]. Tukey’s outlier test was conducted and outliers were removed (IQR = ±1.5), and then a two-tailed heteroscedastic Student’s *t*-test (*p*-value ≤ 0.05) was performed. The samples’ fold change (FC) and Log2 fold change (Log2FC) were then calculated for determining up/down regulation of proteins. The mass spectrometry proteomics data have been deposited to the ProteomeXchange Consortium (http://proteomecentral.proteomexchange.org, accessed on 27 April 2024) via the PRIDE partner repository [36] with the dataset identifier PXD.

Principal Component Analysis (PCA). PCA plots were created using Perseus 1.6.15.0 software (Max Planck Institute of Biochemistry, Planegg, Germany) to aid in interpreting protein quantification and visualizing high-dimensional ‘omics data. The samples were transformed so that each has a mean of 0 and standard deviation of 1. In doing so, it adjusted all samples to be on the same scale by equally weighting them against each other, which allowed for identification of principal components in large datasets. Normalized protein values and associated proteins were input into Perseus and principal components (PC) were calculated through the software. PC1 (*x*-axis) and PC2 (*y*-axis) were utilized in every comparison. Initially, male and female samples were compared for each treatment to determine if there were sex differences. No significant separation was observed, so all samples (male and female) in each treatment were combined for comparison.

Heat Map Generation. Heat maps were generated by GraphPad Prism v9.3.1 (La Jolla, CA, USA), with red boxes within the heatmap indicating upregulation and green indicating downregulation, and threshold auto-adjusted to the specific heatmap. Log2FC of proteins associated with the pathways involved in lipid transport, storage and synthesis, xenobiotic metabolism and transport, inflammation, and lipid catabolism were loaded into a grouped table format. A single or double gradient heat map was then created from this data, allowing for the visualization of up/downregulation of specified proteins.

Ingenuity Pathway Analysis (IPA). IPA was used for the mechanistic analysis, integration and interpretation of the proteomic data. The Log2FC and associated *p*-values for each comparison were loaded into the IPA software version 24.0. For core analyses, data was loaded in the flexible file format, with column headers and an unspecified array platform selected. Default analysis settings were used, interaction networks included 35 molecules per network and 25 networks per analysis, and miRNA Confidence was set to experimentally observed only. Individual analyses were generated for each comparison, and analyses assessing all comparisons sorted by either or: SD, HFD, and the PFAS Mix were combined to look at overarching pathways and upstream regulators between treatments. The workflow for sample preparation and detection and analysis workflow is depicted in Supplementary Figure S1.

## 3. Results

### 3.1. Visualization of Global Proteome

**Figure 2:**
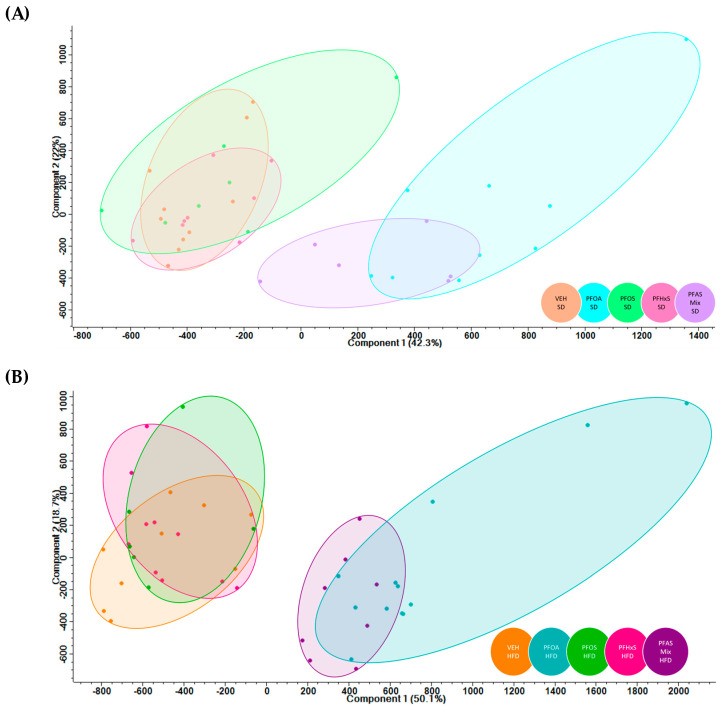
PFOA and PFAS Mix treatments (both SD and HFD) cluster separately from VEH, PFOS and PFHxS (both SD and HFD) treatment groups for offspring liver. (**A**) Standard Diet. (**B**) High-fat Diet.

A volcano plot of differentially expressed proteins (DEPs) (in red) was generated using the Spectronaut^TM^ software and illustrated in Supplementary Figure S2. Overall, there were 1818 DEPs (*p* < 0.05). In dams fed the SD or the HFD, PFOA and PFAS mix, and produced a proteomic expression signature that was distinct from the other treatment groups (Figure 2A,B). PFOA and PFAS Mix treatments produced proteome expression profiles that preferentially clustered together, whereas VEH, PFOS and PFHxS treatment groups had proteome signatures that clustered together. This indicated that PFOA and PFAS Mix samples and VEH, PFOS and PFHxS samples were likely to have overlapping differentially expressed proteins and/or common pathways utilized within their respective clustering.

**Figure 3:**
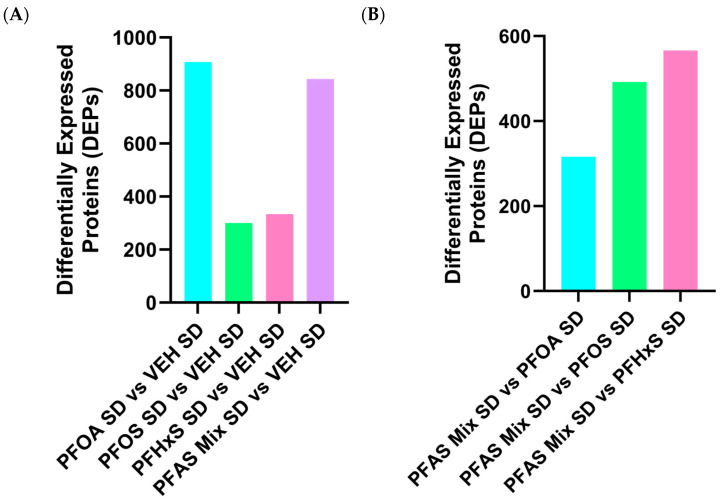

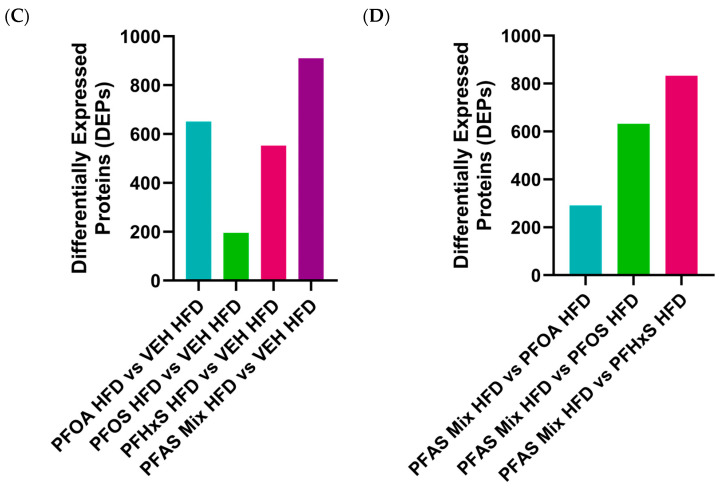
Differentially expressed proteins within each comparison. (**A**) PFAS Treatment* SD compared to VEH SD, (**B**) PFAS Mix SD compared to PFAS Treatment* SD, (**C**) PFAS Treatment* HFD compared to VEH HFD, (**D**) PFAS Mix HFD compared to PFAS Treatment* HFD. * Refers to treatment with PFOA, PFOS or PFHxS.

The PFOA SD versus VEH SD and PFAS Mix SD versus VEH SD comparisons visualized in Figure 3A both show a large sum of differentially expressed protein (DEPs) observed, as compared to the PFOS SD versus VEH SD and PFHxS SD versus VEH SD comparisons. PFOA SD versus VEH SD had 978 DEPs, PFOS SD versus VEH SD resulted in 356 DEPs, PFHxS SD versus VEH SD had 378 DEPs, and the PFAS Mix SD versus VEH SD revealed 879 DEPs. Figure 3B compares the individual PFAS within the PFAS mixture in mice fed a SD, with PFHxS SD having the highest number of proteins commonly observed, followed by PFOS SD, then PFOA SD. A similar trend is observed in Figure 3A, with PFOA and the PFAS Mix observed in Figure 3C looking into the HFD more specifically. The PFAS Mix HFD versus VEH HFD had 926 DEPs, PFOA HFD versus VEH HFD had 750 DEPs, PFOS HFD versus VEH HFD treatment had 205 DEPs, and the PFHxS HFD versus VEH HFD had 584 DEPs. Investigating dams fed a HFD is shown in Figure 3D, which examines the contribution of the individual PFAS within the PFAS mixture and reveals that the PFHxS HFD offspring had the highest number of proteins altered, followed by PFOS HFD, then PFOA HFD.

### 3.2. Effect of PFAS Treatment on the Neonatal Liver Proteome in Dams Fed a Standard Chow Diet

**Figure 4:**
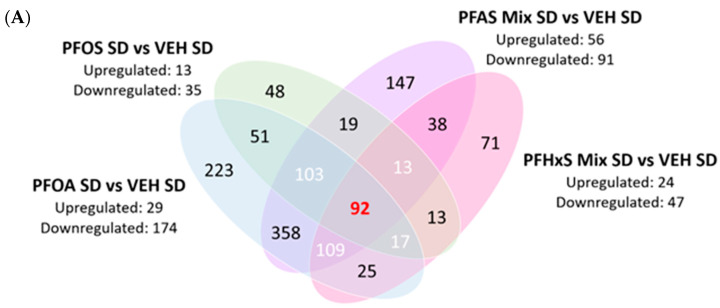

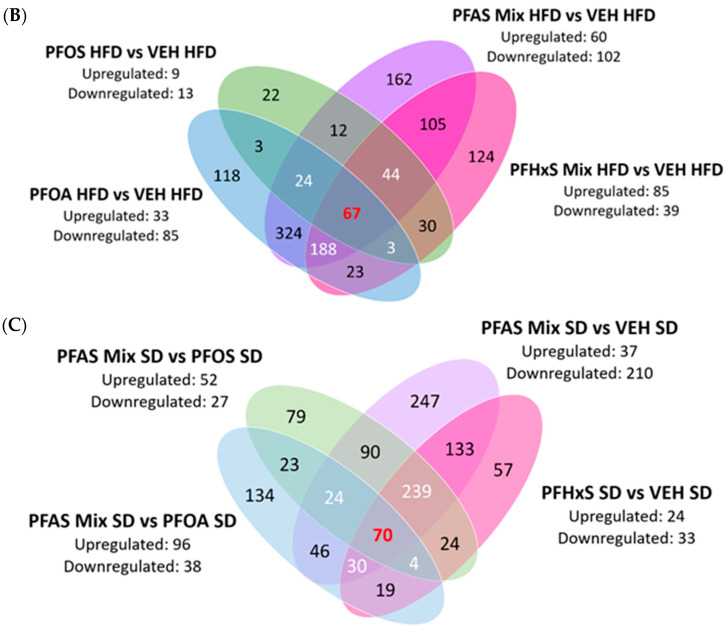
Individual and shared differentially expressed proteins (DEPs) among treatment comparisons. The Venn diagrams were created by Log2 transforming the treatment comparison fold changes of PND21 pups, and then further filtering out all insignificant Log2FC values (*p* < 0.05). Each Venn diagram aims to elucidate both exclusive and commonly expressed significant proteins between comparisons. (**A**) PFAS treatment* SD versus VEH SD, (**B**) PFAS treatment* HFD vs. VEH HFD, and (**C**) PFAS Mix SD vs. PFAS treatment* SD. * PFAS treatment included: PFOA, PFOS, PFHxS.

Livers from PND21 offspring from dams administered a standard diet, PFAS treatment SD versus VEH SD, focusing on the effects of individual PFAS, is visualized in Figure 4A. There were 92 commonly expressed proteins between all comparisons, and the top five upregulated were: Acyl-coenzyme A thioesterase 1 (Acot1)—which catalyzes the hydrolysis into coenzyme A (Coash); free fatty acids from acyl-CoAs, Carboxylesterase 1F (Ces1f)—which participates in the detoxification of foreign bodies; 2,4-dienoyl-CoA reductase [(3E)-enoyl-CoA-producing] (Decr1)—which is involved in fatty acid beta-oxidation; Medium-chain specific acyl-CoA dehydrogenase (Acadm)—which catalyzes step one of mitochondrial fatty acid beta-oxidation; and Acyl-Coenzyme A dehydrogenase family, member 12 (Acad12)—which plays a role in oxidoreductase activity.

#### 3.2.1. Effect of Individual PFAS Treatment (PFOA, PFOS, PFHxS) on the Neonatal Liver Proteome in Mice Fed a Standard Diet

PFOA administration to dams fed the SD resulted in 223 DEPs, PFOS exposure revealed 48 DEPs, and PFHxS had 71 DEPs in livers of PND21 pups (Figure 4A). PFOA exposure modulated pathways involved in immune and metabolic processes, and amino acid metabolism and lipid transport and assembly. The top upregulated protein seen with PFOA exposure is Cathepsin E (Ctse), which plays a role in activation-induced lymphocyte depletion and was upregulated 1.75-fold. PFOA exposure downregulated Alanine aminotransferase 2 (Gpt2), a key intermediate protein in amino acid metabolism, and was observed to be downregulated 1.10-fold. PFOS exposure caused alterations in pathways involved in glucuronidation and elimination, energy regulation, amino acid catabolism, and protein interactions. PFOS caused moderate upregulation, 0.64-fold, of UDP-glucuronosyltransferase 1–6 (Ugt1a6), which plays a role in xenobiotic glucuronidation. Conversely, PFOS downregulated Threonine synthase-like 2 (Thnsl2) 0.68-fold, which is a protein involved in the serine family amino acid catabolic process. PFHxS exposure modified pathways involved in hepatic function, inflammation, lipogenesis, and protein binding. PFHxS caused upregulation, 0.89-fold, of Adenylosuccinate lyase (Adsl), which is involved in the AMP biosynthetic process. Alternatively, PFHxS caused downregulation of Cytochrome p450 2a12 (Cyp2a12), which is involved in arachidonic acid epoxygenase activity, and was downregulated 2.76-fold. The proteins highlighted above are the top up/downregulated proteins unique to each individual PFAS, and are further elaborated in Table 1 and Table 2. Venn diagrams highlighting the COPs of individual PFAS (PFOA, PFOS, PFHxS) and the mixture in dams fed a SD or HFD can be found in Supplementary Figure S3.

#### 3.2.2. Common DEPs between PFOA SD and PFAS Mix SD Treatments Highlight Concordance between Treatments

PFOA and PFAS Mix comparisons in Figure 4A reveal 358 DEPs. The top three are differentially expressed: Acyl-coenzyme A thioesterase 2 (Acot2), which is involved in the fatty acid metabolic process, was upregulated 4.03-fold and 3.69-fold, respectively. Peroxisomal bifunctional enzyme (Ehhadh) is involved in fatty acid beta oxidation, and was upregulated 3.89-fold and 3.45-fold, respectively. Cytochrome p450 2B19 (Cyp2b19), which oxidizes steroids, fatty acids, and xenobiotics, was downregulated 0.78-fold and 1.13-fold, respectively.

#### 3.2.3. DEPs Shared among Maternal PFOA SD, PFHxS SD, and PFAS Mix SD Exposures in the PND21 Neonatal Liver Proteome

At the intersection of PFOA SD versus VEH SD, PFOS SD versus VEH SD, PFHxS SD versus VEH SD, and PFAS Mix SD versus VEH SD, there were 92 commonly expressed DEPs (Figure 4A). The top five modified proteins are: Cytochrome p450 4a14 (Cyp4a14), Cytochrome p450 2b10 (Cyp2b10), Aldehyde dehydrogenase family 3 member a2 (Aldh3a2), Cytochrome p450 4a12a (Cyp4a12a), and Retinal dehydrogenase 1 (Aldh1a1). Cyp4a14 is involved in the metabolism of fatty acids and was upregulated 3.26-fold, 1.08-fold, and 2.94-fold, respectively. Cyp2b10, which plays a role in the oxidation of steroids, fatty acids and xenobiotics, was upregulated 2.59-fold, 1.22-fold, and 2.84-fold, respectively. Aldh3a2 is responsible for the oxidation of medium and long chain aliphatic aldehydes to fatty acids, and was upregulated 2.32-fold, 0.41-fold, and 1.91-fold, respectively. Cyp4a12a is involved in the metabolism of fatty acids and was upregulated 2.10-fold, 0.63-fold, and 1.85-fold, respectively. Aldh1a1 which, by oxidation, converts retinaldehydes into retinoic acid, was upregulated 1.84-fold, 0.68-fold, and 1.69-fold, respectively.

### 3.3. Combinatorial Effect of the PFAS Treatment and HFD on the Neonatal Liver Proteome

Focusing on diet-dependent effects in combination with PFAS administration Figure 4B displays comparisons between PFAS treatment HFD and VEH HFD. There were 67 commonly expressed proteins shared between all comparisons, and the top three upregulated are Acot2, Cyp4a14, and Carboxylesterase 1d (Ces1d), which is involved in cholesterol and acyl-CoA metabolism. The top three decreased proteins were Cytochrome p450 2c39 (Cyp2c39), which plays a role in the arachidonic acid metabolic process, 2-oxo-4-hydroxy-4-carboxy-5-ureidoimidazoline decarboxylase (Urad), involved in chemical reactions and pathways resulting in the breakdown of adenosine, and Coenzyme q5 (Coq5), which is involved in methylation and ubiquinone biosynthetic processes.

#### 3.3.1. DEPs Shared among Maternal HFD and PFOA SD; PFHxS SD, and PFAS Mix SD Exposures in the PND21 Neonatal Liver Proteome

In dams fed the HFD, PFOA induced 188 DEPs, PFOS induced 22, and PFHxS induced 124 (Figure 4B). PFOA exposure modulated pathways involved in lipid and steroid metabolism, protein phosphorylation function, arachidonic acid and glutathione metabolic processes, and oxidative demethylation. PFOA exposure increased NADPH-dependent 3-keto-steroid reductase (Hsd3b5), which is mostly involved in the formation of steroids, and it was upregulated 0.83-fold with a HFD. PFOA exposure and HFD decreased Cytochrome p450 2d9 (Cyp2d9) 1.62-fold, which is a protein that plays a role in the arachidonic acid metabolic process. PFOS exposure caused alterations in pathways involved in mitochondrial electron transport, cellular amino acid metabolic processes, apoptotic processes, cholesterol and carbohydrate metabolism, and oxidoreductase activity. PFOS increased NADH-ubiquinone oxidoreductase chain 2 (Mt-nd2), which is a protein responsible for electron transfer from NADH to ubiquinone during oxidative phosphorylation, by 0.65-fold. PFOS altered 1,5-anhydro-D-fructose reductase (Ec:1.1.1.263), which is responsible for the catalysis of redox reactions, and was observed to be decreased by 1.25-fold. PFHxS exposure modified pathways involved in protein and organic anion transport, oxidative demethylation, fatty acid alpha-oxidation, calcium ion binding, and cholesterol homeostasis. PFHxS caused increased Selenium-binding protein 2 (Slenbp2), which is involved in detecting reactive xenobiotics in the cytoplasm, by 4.66-fold. Alternatively, PFHxS decreased Reticulocalbin-2 (Rcn2), which is involved in hepatic growth factor signaling, 0.77-fold. The proteins highlighted above are the top up/downregulated proteins unique to each individual PFAS and are further elaborated upon in Table 1 and Table 2. Venn diagrams highlighting the COPs of individual PFAS (PFOA, PFOS, PFHxS) and the mixture in dams fed a SD or HFD can be found in Supplementary Figure S3.

#### 3.3.2. Shared DEPs at the Intersection of Maternal PFOA HFD, PFHxS HFD; and PFAS Mix HFD Treatments in the Neonatal Liver Proteome

As demonstrated in Figure 4B, PFOA, PFHxS, and PFAS Mix shared a larger collection of commonly expressed proteins. PFOA HFD versus VEH HFD, PFHxS HFD versus VEH HFD, and PFAS Mixture HFD versus VEH HFD groups had 188 shared DEPs. The top five modified proteins were: Acot1, Cyp2b10, Ces1f, Cytochrome p450 4a10 (Cyp4a10), and Aldh3a2. Acot1 was increased 4.78-fold, 0.98-fold, and 4.68-fold, respectively. Cyp2b10 was upregulated 3.64-fold, 0.95-fold, and 4.50-fold, respectively. Ces1f, was upregulated 3.22-fold, 1.65-fold, and 3.41-fold, respectively. Cyp4a10 is responsible for the metabolism of fatty acids, and was upregulated 2.36-fold, 1.38-fold, and 2.88-fold, respectively. Aldh3a2 was upregulated 2.41-fold, 0.50-fold, and 2.80-fold, respectively.

### 3.4. Contributions of Individual PFAS within the PFAS Mixture

Comparison of the PFAS Mix SD versus PFAS treatment* SD, focusing on structure and cumulative effects of a PFAS mixture with its individual counterpart, is visualized in Figure 4C. There were 70 shared DEPs among all comparisons (PFAS Mix SD versus VEH SD, PFAS Mix SD versus PFOA SD, PFAS Mix SD versus PFOS SD, PFAS Mix SD versus PFHxS SD). The top five altered are: Aldh3a2, Epoxide hydrolase 1 (Ephx1), Leukocyte elastase inhibitor A (Serpinb1a), Ethanolamine phosphate phospholyase (Etnppl), and Acss3. Aldh3a2 was upregulated in the PFAS Mix SD versus VEH SD (1.91-fold), PFAS Mix SD versus PFOS SD (1.58-fold), PFAS Mix SD versus PFHxS SD (1.50-fold) comparisons, however, was moderately downregulated in the PFAS Mix SD versus PFOA SD (0.40-fold) comparison. Ephx1 is responsible for the metabolism of endogenous lipids, and also followed a similar trend as Aldh3a2 with the PFAS Mix SD versus PFOA SD comparison (0.29-fold), marginally downregulated. The other three comparisons being upregulated with PFAS Mix SD versus VEH SD (1.31-fold), PFAS Mix SD versus PFOS SD (1.55-fold), PFAS Mix SD versus PFHxS SD (1.14-fold). Serpinb1a, which deals with cellular homeostasis and inflammatory responses also followed suit with slight downregulation in the PFAS Mix SD versus PFOA SD comparison (-0.94-fold). The other three comparisons being upregulated with PFAS Mix SD versus VEH SD (1.79-fold), PFAS Mix SD versus PFOS SD (2.08-fold), PFAS Mix SD versus PFHxS SD (1.39-fold). Etnppl is involved in the metabolism of proteins, lipids, and carbohydrates and all treatments are upregulated in the comparisons: PFAS Mix SD versus VEH SD (1.31-fold), PFAS Mix SD versus PFOA SD (0.99-fold), PFAS Mix SD versus PFOS SD (0.67-fold), PFAS Mix SD versus PFHxS SD (0.52-fold).

### 3.5. Pathway Modulation among Diet and Mixture-Specific Comparisons

#### 3.5.1. Lipid, Xenobiotic and Inflammation Pathway Modulation within SD Comparisons

**Figure 5:**
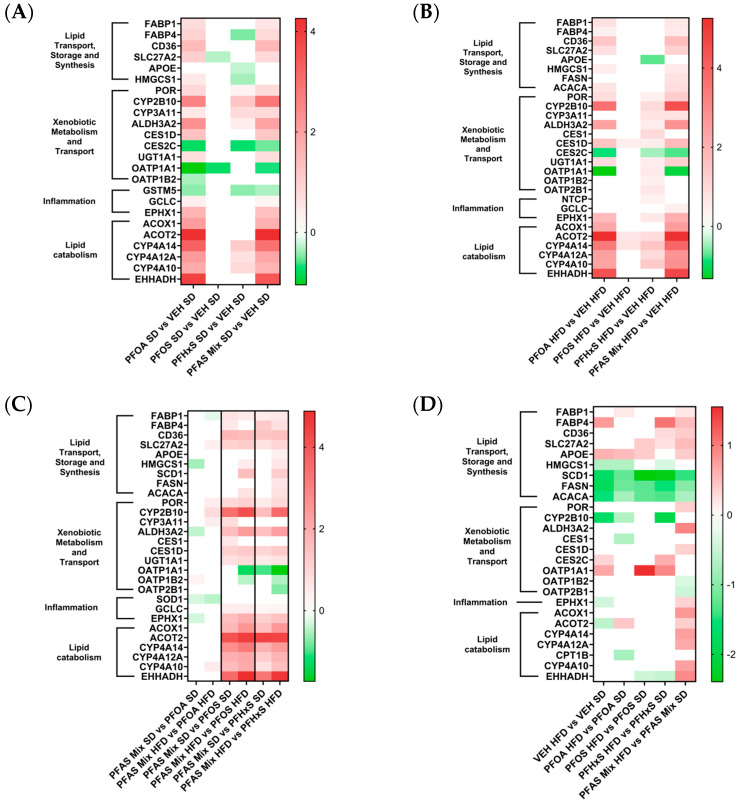
Evaluation of PFOA, PFOS, PFHxS; and a PFAS Mixture-induced modulation of lipid metabolism and transport, xenobiotic metabolism and transport, and lipid catabolism pathways in the neonatal liver. Heatmaps (**A**–**D**) represent a Log2 transformation of comparison fold changes, by filtering out of all insignificant Log2FC values (*p* < 0.05).

The top proteins differentially expressed between mice exposed to PFAS mix versus VEH from dams fed the SD is highlighted in Figure 5A, and focuses on lipid transport, storage and synthesis, xenobiotic metabolism and transport, inflammation, and lipid catabolism. Of the modulated proteins, the most increased proteins include: Acot2, Ehhadh, Cyp4a14, and Cyp2b10. The most decreased proteins include: Oatp1a1, Ces2c, and Fabp4. PFOA and the PFAS Mixture share a similar signature of protein modulation, regarding protein expression within these pathways, whereas the PFOS and PFHxS effect was less robust. Detailed descriptions of each protein can be found in Table 3.

#### 3.5.2. Lipid, Xenobiotic and Inflammation Pathway Modulation within HFD Comparisons

The top proteins modulated within the PFAS Treatment HFD versus VEH HFD are illustrated in Figure 5B, with a focus on lipid transport, storage and synthesis, xenobiotic metabolism and transport, inflammation, and lipid catabolism. Upregulated proteins include Acot2, Ehhadh, Cyp4a14, and Cyp2b10. The most include Oatp1a1, Ces2c, and Apoe. Similar to the trend observed in Figure 5A, there was a similar expression signature for PFOA and the PFAS Mixture comparisons existing in livers from dams from HFD. Additional proteins not found to be modulated in the SD comparisons include Fasn, Acaca, Ces1, Oatp2b1, and Ntcp. Detailed descriptions of each protein can be found in Table 3.

#### 3.5.3. Lipid, Xenobiotic and Inflammation Pathway Modulation within the PFAS Mixture

Figure 5C depicts the top proteins modulated within the PFAS Treatment comparing SD/HFD versus VEH HFD/SD for lipid transport, storage and synthesis, xenobiotic metabolism and transport, inflammation, and lipid catabolism. The top increased proteins included Cyp2b10, Acot2, Ehhadh, Cyp4a14, and Aldh3a2. The proteins with most decreased expression included Oatp1a1, Oatp2b1, and Hmgcs1. When evaluating the contribution of an individual PFAS to the PFAS Mixture, the data revealed that PFOA SD or HFD in the mixture has notable differences from PFOS SD/HFD and PFHxS SD/HFD. PFOA SD/HFD and the PFAS Mixture SD/HFD treatments caused similar protein expression profiles in neonatal liver.

#### 3.5.4. Synthesis and Oxidation of Lipid Pathway Modulation within the PFAS Mixture

**Figure 6:**
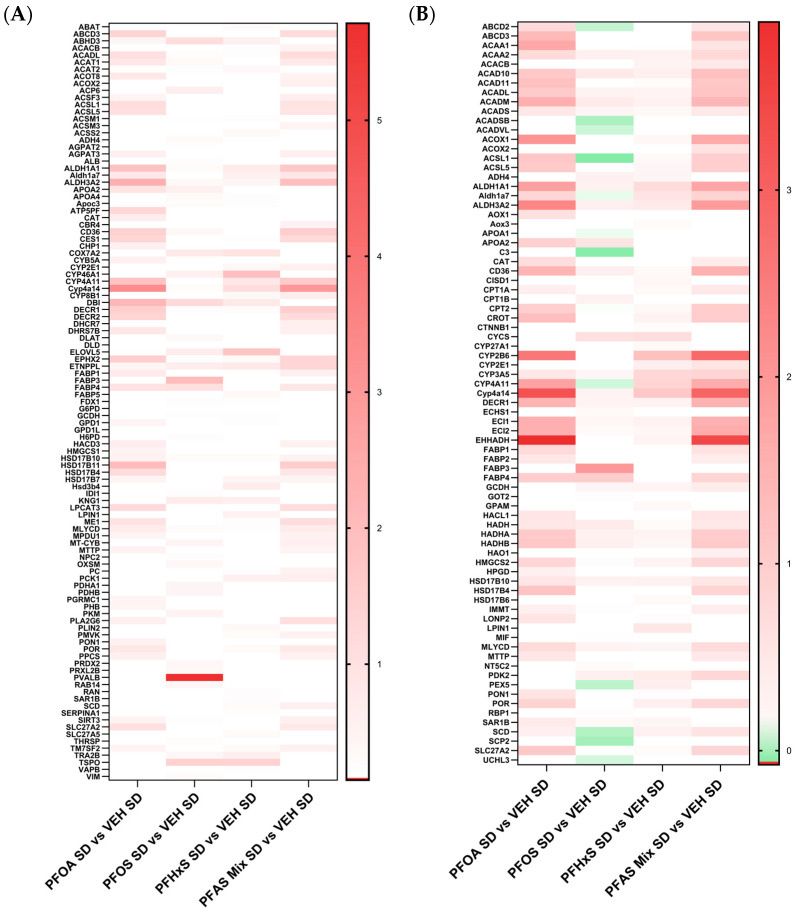
Expression of proteins in lipid synthesis and oxidation of pathways. Differential expression of proteins contributing to the (**A**) synthesis of lipids and (**B**) oxidation of lipids were identified through IPA disease and biological function analyses. The heat maps were created by Log2 transforming the standard diet treatment comparison fold changes of PND21 pup livers. The treatment comparisons consist of: PFOA SD and VEH SD, PFOS SD vs. VEH SD, PFHxS SD vs. VEH SD, and PFAS Mix SD vs. VEH SD.

IPA analysis revealed activation of lipid synthesis and oxidation pathways (Figure 6A,B). Log2 fold change values of the specified proteins within the pathways were used to create the heatmaps in Figure 6, focusing on PFOA SD versus VEH SD, PFOS SD versus VEH SD, PFHxS SD versus VEH SD, and PFAS Mix SD versus VEH SD comparisons. The PFOA SD versus VEH SD and PFAS Mix SD versus VEH SD comparisons share common upregulation signatures. Conversely, the PFOS SD versus VEH SD and PFHxS SD versus VEH SD share commonly upregulated proteins within these two pathways. The top altered proteins within these pathways included Parvalbumin (Pvalb), Cyp4a14, Cytochrome p450 4a11 (Cyp4a11), Aldh1a1, Aldh3a2, Fabp3, Cd36, and Ephx2. Overall, the PFOA, PFOS, PFHxS and PFAS Mixture exposure during gestation and lactation predominantly upregulated proteins involved in pathways related to the synthesis and oxidation of lipids.

#### 3.5.5. Proteins Modulated in Pathways Related to Liver Damage within the PFAS Mixture

**Figure 7:**
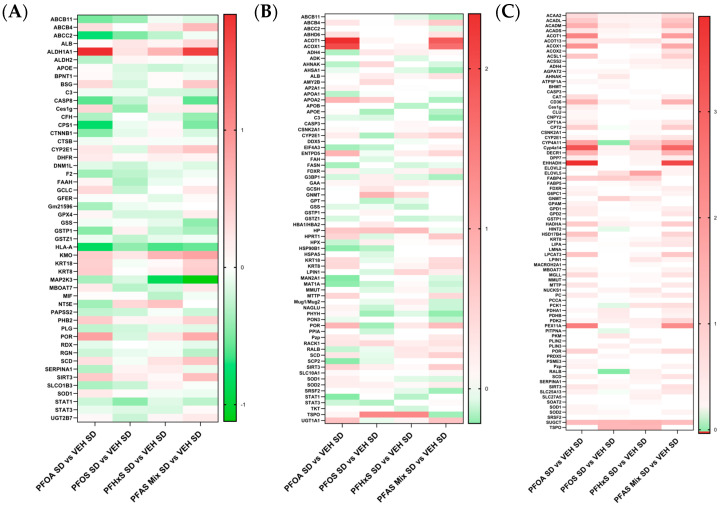
Proteins modulated in pathways related to liver damage. Proteins involved in (**A**) liver damage, (**B**) inflammation of liver, and (**C**) hepatic steatosis disease and biological function pathways were identified through IPA analyses. The heat maps were created by Log2 transforming the standard diet treatment comparison fold changes of PND21 pup livers. The treatment comparisons consist of PFOA SD vs. VEH SD, PFOS SD vs. VEH SD, PFHxS SD vs. VEH SD, and PFAS Mix SD vs. VEH SD.

IPA analyses of disease and biological function pathways revealed modulation related to liver damage (Figure 7A), liver inflammation (Figure 7B) and hepatic steatosis (Figure 7C). The heat maps in Figure 7 display Log2 fold change values within the PFOA SD versus VEH SD, PFOS SD versus VEH SD, PFHxS SD versus VEH SD, and PFAS Mix SD versus VEH SD comparisons. Downregulation of proteins is predominantly seen across all comparisons in Figure 7A, with the exception of a few proteins commonly upregulated: Aldh1a1, Kynurenine 3-monooxygenase (Kmo), and Cytochrome p450 oxidoreductase (Por). The top proteins downregulated include Mitogen-activated protein kinase 3 (Map2k3), Carbamoyl-phosphate synthetase 1 (Cps1), and ATP-binding cassette, sub-family C (Cftr/Mrp), member 2 (Abcc2). Liver inflammation shows a broad distribution of upregulated and weakly downregulated proteins (Figure 7B). The top upregulated proteins within liver inflammation include Acot1, Acox1, Translocator protein (Tspo), and Haptoglobin (Hp). The top downregulated proteins include Alpha-mannosidase 2 (Man2a1), Fasn, Glutathione synthetase (Gss), and Signal transducer and activator of transcription 1 (Stat1). Proteins involved in hepatic steatosis progression are predominantly upregulated, with a few proteins downregulated in the PFOS SD versus VEH SD comparison, as visualized in Figure 7C. The top upregulated proteins include: Ehhadh, Cyp4a14, Acot1, Acox1, and Succinate-hydroxymethylglutarate CoA-transferase (Sugct). Overall, the proteins involved in the pathway relating to liver inflammation were more equally distributed between up and downregulation, whereas liver damage was predominantly downregulated, and hepatic steatosis upregulated.

#### 3.5.6. Proteins Modulated in Pathways Related to Fatty Acid Metabolism, Oxidation and Transport within the PFAS Mixture

**Figure 8:**
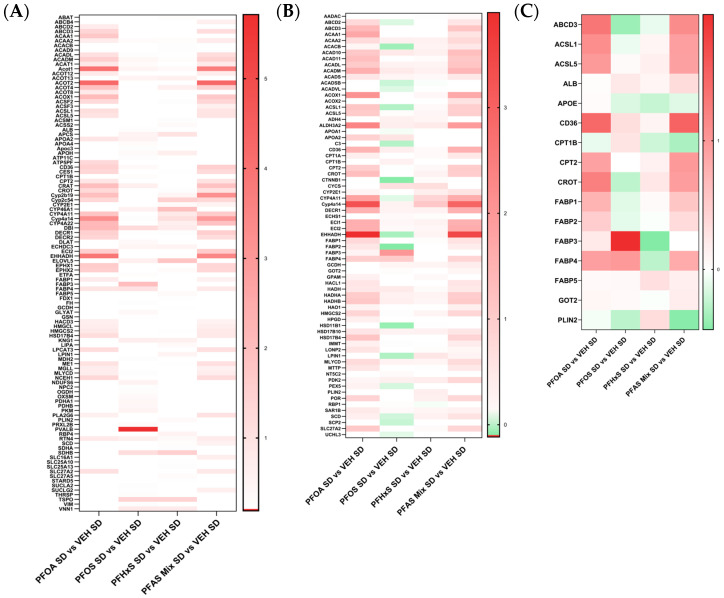
Proteins modulated in pathways related to fatty acid metabolism and transport. Proteins involved in (**A**) fatty acid metabolism, (**B**) oxidation of fatty acids, and (**C**) transport of long chain fatty acids were identified by IPA’s disease and biological function pathway analysis. Heat maps represent Log2 transformation of comparisons of PFAS treatment versus VEH control. The comparisons between treatments were as follows: PFOA SD vs. VEH SD, PFOS SD vs. VEH SD, PFHxS SD vs. VEH SD, and PFAS Mix SD vs. VEH SD.

The heatmaps in Figure 8 display the Log2 fold change values within the PFOA SD versus VEH SD, PFOS SD versus VEH SD, PFHxS SD versus VEH SD, and PFAS Mix SD versus VEH SD comparisons. IPA analyses of disease and biological function pathways revealed protein changes related to fatty acid metabolism (Figure 8A), fatty acid oxidation (Figure 8B) and transport of long chain fatty acids (Figure 8C). The fatty acid metabolism pathway was completely upregulated as observed in Figure 8A; the proteins with most increased expression were Acot1, Acot2, Ehhadh, and Pvalb. Protein modulation within the oxidation of fatty acids was predominantly upregulated, with only the PFOS SD versus VEH SD showing very slight downregulation of select proteins, including Acacb, Ctnnb1, and Fabp2 (Figure 8B). The proteins with the highest increase in expression across all comparisons were Acox1, Aldh3a2, and Cyp4a14. A handful of proteins involved in the transport of long chain fatty acids, showing both increased and decreased expression, are depicted in Figure 8C. The top proteins upregulated across all comparisons were Cd36 and Carnitine O-palmitoyltransferase 2 (Cpt2); the protein most downregulated across all comparisons was ApoE. Overall, the PFOA SD versus VEH SD and PFAS Mix SD versus VEH SD comparisons exhibited similar protein signatures, whereas the PFOS SD versus VEH SD and PFHxS SD versus VEH SD comparisons exhibited similar patterns of protein expression related to fatty acid metabolism, oxidation and transport.

## 4. Discussion

The purpose of this work is to evaluate how administration of PFOA, PFOS, PFHxS, or a 1:1:1 PFAS mixture affected the proteome in livers of offspring at PND21. Additionally, the work herein also explored effect of maternal diet on liver proteome outcomes. SWATH–MS was utilized alongside IPA to generate a framework for investigating phenotypic and functional outcomes within the context of established biological structures/systems. This approach has been used by our group to quantify changes in the liver and adipocyte proteome of adult male mice exposed to PFOS, PFHxS, and PFNA, but has not been described for effects on offspring [20,22,37,38]. Through investigating the similarities and differences observed between treatment/diet comparisons, distinctions between PFAS are revealed.

PFOA, PFOS, and PFHxS modified the proteome of livers of pups exposed via dams during gestation and lactation, and the PFAS mixture (both SD and HFD) exerted a more robust modulation and activation than individual PFAS. Examining the contributions of PFOA, PFOS, and PFHxS individually compared to the PFAS mixture signature overall, PFOA was revealed to be a driver of the effects in the presence of PFOS and PFHxS. This trend was first revealed in Figure 2, which illustrates the preferential clustering of PFOA and the PFAS mixture from VEH, PFOS, and PFHxS, independent of diet. The PCA plots depict the protein signatures of each individual pup liver by adjusting all the samples to be on the same scale by equally weighting them against each other. Thus, allowing for a baseline view of the distinct similarities/differences seen within/between treatments, this trend was supported upon further data analysis. In examining Figure 3B,D, increased DEPs are observed for PFOS and PFHxS within the PFAS mixture, as compared to their respective VEH controls, for both SD and HFD. This trend is reiterated in Figure 4C, where 239 proteins were shared between the PFOS SD and PFAS Mix SD, PFHxS SD and PFAS Mix SD, and VEH SD and PFAS Mix SD comparisons. Figure 3A,C suggest that PFOA and the PFAS mixture activate a similar number of proteins in dams fed a SD or HFD, respectively. This observation is also demonstrated in Figure 4A,B, where PFOA vs. VEH and PFAS Mix vs. VEH comparisons had 358 (SD) and 324 (HFD) proteins commonly shared.

A clear visual representation of the concordance between PFOA and the PFAS mixture is highlighted in Figure 5, Figure 6, Figure 7 and Figure 8, with heat maps displaying proteins involved in lipid transport, storage, oxidation, and synthesis, xenobiotic metabolism and transport, liver damage and inflammation, and fatty acid metabolism, oxidation and transport. The PFOA SD/HFD versus VEH SD/HFD comparisons in these figures parallel the proteomic signatures of the PFAS Mixture SD/HFD versus VEH SD/HFD. Both treatment groups had similar levels of modulation and activation, with it being slightly higher in the PFAS mixture. PFOA differentially behaves alone versus within the PFAS mixture, with individual PFOA signatures opposing what is witnessed when PFOA is isolated from the PFAS mixture treatments, independent of diet. Conversely, the PFOS SD/HFD versus VEH SD/HFD and PFHxS SD/HFD versus VEH SD/HFD share a similar proteomic signature, both showing weak modulation and activation, and slight disagreement to PFOA and the PFAS Mixture, regardless of diet. PFOS SD/HFD and PFHxS SD/HFD signatures within the PFAS mixture mirror the signature of PFOA SD/HFD versus VEH SD/HFD individually. This suggests that PFOA dominates the overall modulation observed by the PFAS mixture, and potentiates any effects seen by PFOS and PFHxS within the mixture.

Additional analysis from this same study revealed that PFOA, PFOS, and PFHxS administered to the dam resulted in measurable concentrations in pup livers: 11.73 μg/mL (PFOA SD), 11.47 μg/mL (PFOA/Mix SD), 17.51 μg/mL (PFOA HFD), 15.21 μg/mL (PFOA/Mix HFD), 0.61 μg/mL (PFOS SD), 0.28 μg/mL (PFOS/Mix SD), 0.46 μg/mL (PFOS HFD), 0.39 μg/mL (PFOS/Mix HFD), 27.97 μg/mL (PFHxS SD), 24.41 μg/mL (PFHxS/Mix SD), 24.1 μg/mL (PFHxS HFD), 25.67 μg/mL (PFHxS/Mix HFD) [31]. The concentrations measured in the pup livers may contribute to the DEP signatures observed. PFOS SD and HFD treatment groups had the lowest number of DEPs, and the lowest measured liver concentrations—suggesting that liver PFOS concentration was related to protein expression change. PFHxS SD and HFD had the highest measured liver concentrations, and the second highest number of DEPs. The number of PFHxS SD DEPs was similar to the number of PFOS SD DEPs, and PFHxS HFD altered a similar number of DEPs to PFOA HFD. PFOA SD and HFD had tissue concentrations between PFOS SD/HFD and PFHxS SD/HFD, but PFOA treatment resulted in the most DEPs for both SD and HFD. PFOA, at a lower measured concentration than PFHxS, was able to more robustly alter the pup liver proteome, suggesting PFOA may be more potent than PFHxS. The PFAS mixture administered to dams had distinct effects on pup liver weights and lipids, compared to individually administered PFAS An increase in liver lipids, elevated serum ALT, and altered serum leptin was observed in the dams. [31]. Some limitations to the study lie within the number of samples within the PFOS HFD (*n* = 6) and PFAS Mix SD (*n* = 6) treatments. Of the five dams per treatment/diet, there were some dams dosed with PFOS SD and the PFAS Mix SD that did not get pregnant and give birth, and/or had difficult births and spontaneously aborted their litter, which lowered the “n” per group. Additionally, to further understand the contribution of each PFAS to the mixture, it would be advantageous to isolate combinations of PFOA + PFOS, PFOA + PFHxS, and PFHxS + PFOS. This would allow us to further tease out which PFAS is dominating the overall protein signature between the two mixtures and more thoroughly investigate how, and to what degree, PFOA may be potentiating PFOS and PFHxS within a mixture.

The top DEPs across all comparisons are predominantly upregulated and have been previously reported as such in literature looking at the effects of PFAS exposure on proteomic liver changes [7,9,10,12,14,19,20,21,22,37,38]. Of those commonly upregulated, Acox1, Acot1, and Acot2 are all involved in lipid catabolism, lipid synthesis, liver inflammation, and hepatic steatosis. Ehhadh and Cyp4a14 are both involved in lipid catabolism, lipid synthesis, and hepatic steatosis. Cd36 and Slc27a2 are both involved in lipid synthesis and oxidation, and fatty acid metabolism, oxidation; and only Cd36 is involved in fatty acid transport and hepatic steatosis. Aldh3a2 and Cyp2b10 (upregulated) and Oatp1a1 and Ces2c (downregulated) are all involved in xenobiotic metabolism and transport. Aldh3a2 is additionally involved in lipid synthesis and oxidation and fatty acid oxidation. Although there are few proteins within the highlighted pathways that are downregulated, the same trend observed in the commonly upregulated proteins still however holds. Across all treatments and diets ApoE is downregulated in the transport of long chain fatty acids and liver damage and inflammation. Stat1 is downregulated in the liver damage and inflammation pathways. All of these proteins have been reported in both SD and HFD comparisons of single PFOA, PFOS and PFHxS treatments and the PFAS mixture. The proteomic changes revealed in this data are consistent with the observed lipid deposition in the pup livers, described previously [31].

Our laboratory previously investigated PFOS effects on the adult hepatic proteome in 6-week-old male C57BL/6J mice administered PFOS via the diet at about 0.36 mg/kg/day for 10 weeks. PFOS upregulated Acox1 (2-fold), Acot2 (2-fold), Ehhadh (3-fold), Cyp4a14 (4.5-fold), Aldh3a2 (1.5-fold), Cyp2b10 (1-fold), and Oatp1a1 (1-fold) [20]. Many of these previously described upregulated proteins were also observed in pup livers in this current study. Additionally, another study conducted by our laboratory investigated PFOS and PFHxS effect on the adult proteome. There, 10-week-old, male C57BL/6J mice were fed either 0.0003% PFOS or 0.0003% PFHxS in rodent chow for 29 weeks. This caused an upregulation of Acox1 (1.7 and 1.2-fold respectively), Acot2 (2.4 and 1.1-fold respectively), Ehhadh (3.3 and 1.1-fold respectively), Cyp4a14 (2.8 and 2.0-fold respectively), and Aldh3a2 (1.7 and 1.1-fold respectively) [22]. A study utilizing mouse 3T3-L1 fibroblasts was conducted to assess the adipogenicity potential of ten different PFAS (PFBS, PFHxS, PFOS, PFBA, PFHxA, PFHA, PFOA, PFNA, PFDA, and HFPO-DA), and the cellular proteome was analyzed. The 3T3-L1 cells were treated with/without rosiglitazone and the single PFAS at concentrations of 0.25–25 μM or Vehicle (0.1% DMSO). PFOA, PFOS and PFHxS were shown to significantly affect lipid metabolism, nucleic acid metabolism, and cell-to-cell signaling and interaction [38].

In a human health review of PFAS toxicity, it was concluded that there is strong confluence between animal toxicology and histology, and human health data. This supports the notion that PFAS disrupt hepatic metabolism, alter bile acid metabolism, and lead to lipid accumulation [10]. The data presented herein reinforce this conclusion, with the added component of these adverse events caused by an indirect developmental exposure. Another health review, focusing on early life exposures and the latent health outcomes, concluded that PFAS induced adverse outcomes in the placenta through lipid and sterol disruption, oxidative stress and epigenetic alterations [26]. In analyzing the European Human Early-Life Exposome (HELIX) cohort of 1105 mothers and their children, from 2014–2015, a recent metabolome-based review revealed PFOA, PFOS, PFHxS, PFNA, and PFUnDA exposure significantly increased prenatal and child serum levels of branched-chain amino acids (BCAAs) and aromatic amino acids (AAAs). These indicate the activation of amino acid metabolism and lipid metabolism pathways [39]. Elevated circulating levels of BCAAs and AAAs have been associated with NAFLD in children [40].

Few reports exist that describe maternal exposure impact on liver function in general, with even fewer reports regarding impact on the proteome. This novel study established that indirect exposure to the fetus/pup via maternal PFAS intake can alter the liver proteome in the offspring, and predisposes the pups to adverse liver outcomes and altered metabolism. PFOA potentiated the signature of the PFAS mixture and an overall greater modulation of proteins was seen within the mixture. The importance of these findings is key in setting regulations for PFAS’s found in water, clothing and food packaging, etc., and setting concentration level limits for the general population.

## 5. Conclusions

The results indicate that the administration of either PFOA, PFOS, PFHxS or a 1:1:1 PFAS mixture does impart alterations in the offspring’s liver proteome. In comparing individual PFAS exposures to that of the mixture, the mixture resulted in more DEPs than any individual PFAS, with HFD exacerbating the observed mixture effect. Dam exposure to PFOA and the PFAS mixture altered the highest number of proteins in livers of PND21 offspring, as compared to PFOS and PFHxS administration, independent of diet. The heat maps of the top DEPs involved in lipid transport, storage and synthesis, xenobiotic metabolism and transport, inflammation, and lipid catabolism reveal marked differences in up/downregulation when considering PFOA SD, PFOS SD or PFHxS SD individually, compared to when combined as a PFAS SD mixture. Overall, PFOA may drive DEP when in the presence of a mixture in vivo. The data also indicate that gestational and lactational exposure to PFOA, PFOS and PFHxS predisposes pups to liver damage and inflammation, upregulation of the synthesis, oxidation of lipids, upregulation of fatty acid metabolism, and oxidation of fatty acid pathways.

## Figures and Tables

**Table 1:** Top upregulated proteins unique to individual PFAS (PFOA, PFOS, PFHxS) in dams fed a SD and HFD.

Treatment	Log2FC	*p*-Value	Abbreviation	Protein Name	Function
PFOA SD	1.75	0.00479	Ctse	Cathepsin E	Role in activation-induced lymphocyte depletion
	1.40	6.90 × 10^−7^	Gm4952	Glycine N-acyltransferase-like protein	Enables glycine N-acyltransferase activity
PFOS SD	0.64	0.04073	Ugt1a6	UDP-glucuronosyltransferase 1–6	Role in xenobiotic glucuronidation
	0.64	0.03844	Fhit	Bis(5′-adenosyl)-triphosphatase	Involved in the diadenosine triphosphate catabolic process
PFHxS SD	0.89	0.04754	Adsl	Adenylosuccinate lyase	Involved in the AMP biosynthetic process
	0.92	0.04504	Cyp2a5	Cytochrome p450 2a5	Involved in arachidonic acid epoxygenase activity
PFOA HFD	0.83	0.03687	Hsd3b5	NADPH-dependent 3-keto-steroid reductase	Involved in the formation of steroids
	0.53	0.00257	Acot13	Acyl-coenzyme A thioesterase 13	Involved in the lipid metabolic process
PFOS HFD	0.65	0.01482	Mt-nd2	NADH-ubiquinone oxidoreductase chain 2	Responsible for electron transfer during oxidative phosphorylation
	0.57	0.02997	Dpyd	Dihydropyrimidine dehydrogenase	Involved in pyrimidine nucleoside monophosphate catabolic process
PFHxS HFD	4.66	0.03114	Slenbp2	Selenium-binding protein 2	Involved in detecting reactive xenobiotics in the cytoplasm
	1.98	4.51 × 10^−4^	Cyp3a41	Cytochrome p450 3a41	Involved in lipid hydroxylation

**Table 2:** Top downregulated proteins unique to individual PFAS (PFOA, PFOS, PFHxS) in dams fed a SD and HFD.

Treatment	Log2FC	*p*-Value	Abbreviation	Protein Name	Function
PFOA SD	−1.10	1.65 × 10^−6^	Gpt2	Alanine aminotransferase 2	Key intermediate protein in amino acid metabolism
	−0.90	1.05 × 10^−4^	Lpin1	Phosphatidate phosphatase lpin1	Involved in the cellular response to insulin stimulation
PFOS SD	−0.68	0.01353	Thnsl2	Threonine synthase-like 2	Involved in the serine family amino acid catabolic process
	−0.68	0.01542	Ldhb	L-lactate dehydrogenase B chain	Lactate metabolic process
PFHxS SD	−1.05	0.01015	Cyp2a12	Cytochrome p450 2a12	Arachidonic acid epoxygenase activity
	−0.97	0.03609	Atp5pf	ATP synthase-coupling factor 6, mitochondrial	Negative regulation of arachidonic acid secretion
PFOA HFD	−1.62	0.01037	Cyp2d9	Cytochrome p450 2d9	Role in the arachidonic acid metabolic process
	−1.18	0.04546	Cyp3a13	Cytochrome p450 3a13	Aids in removing methyl groups via oxidation
PFOS HFD	−1.25	0.00885	Ec:1.1.1.263	1,5-anhydro-D-fructose reductase	Responsible for the catalysis of redox reactions
	−1.03	0.04074	Apoa2	Apolipoprotein A-II	Role in lipid binding
PFHxS HFD	−0.77	0.00175	Rcn2	Reticulocalbin-2	Involved in hepatic growth factor signaling
	−0.70	0.01179	Apoe	Apolipoprotein E	Regulate plasma lipoprotein metabolism

**Table 3:** Top proteins up/downregulated within Lipid Transport, Storage and Synthesis, Xenobiotic Metabolism and Transport, Inflammation and Lipid catabolism.

Pathway	Abbreviation	Protein Name	Function
Lipid Transport, Storage and Synthesis	Acaca	Acetyl-CoA carboxylase 1	Enzyme that catalyzes the rate-limiting step in fatty acid synthesis
	Apoe	Apolipoprotein E	Lipoprotein-mediated lipid transport
	Cd36	Platelet glycoprotein 4	Involved in long chain fatty acid uptake
	Fabp1	Fatty acid-binding protein 1	Role in fatty acid uptake, transport, and metabolism
	Fabp4	Fatty acid-binding protein 4	Role in fatty acid uptake, transport, and metabolism
	Fasn	Fatty acid synthase	Catalyzes long-chain saturated fatty acids from acetyl-coa and malonyl-coa
	Hmgcs1	Hydroxymethylglutaryl-CoA synthase	Catalyzes the formation of HMG-coa
	Scd1	Acyl-CoA desaturase 1	Involved in fatty acid biosynthesis
	Slc27a2	Very long-chain acyl-CoA synthetase	Catalyzing the formation of fatty acyl-coa
Xenobiotic Metabolism and Transport	Aldh3a2	Aldehyde dehydrogenase family 3 member a2	Catalyzes the oxidation of medium and long-chain aliphatic aldehydes to fatty acids
	Ces1	Liver carboxylesterase 1	Detoxification of xenobiotics
	Ces1d	Carboxylesterase 1d	Metabolism of xenobiotics and of natural substrates
	Ces2c	Acylcarnitine hydrolase	Prostaglandin metabolic process
	Cyp2b10	Cytochrome p450 2b10	Oxidizes steroids, fatty acids, and xenobiotics
	Cyp3a11	Cytochrome p450 3a11	Steroid metabolic process
	Ntcp	Sodium/bile acid cotransporter	Transporter of conjugated bile salts from plasma into the hepatocyte
	Oatp1a1	Organic anion transporting polypeptide 1a1	Mediates the Na+-independent transport of organic anions
	Oatp1b2	Organic anion transporting polypeptide 1b2	Mediates the Na+-independent uptake of organic anions
	Oatp2b1	Organic anion transporting polypeptide 2b1	Mediates the Na+-independent transport of organic anions
	Por	Cytochrome p450 oxidoreductase	Donate electrons directly from NADPH to all microsomal p450 enzymes
	Ugt1a1	UDP-glucuronosyltransferase 1a1	Catalyzes phase II biotransformation reactions in which lipophilic substrates are conjugated with glucuronic acid
Inflammation	Ephx1	Epoxide hydrolase 1	Catalyzes the hydrolysis of arene and aliphatic epoxides
	Gclc	Glutamate-Cysteine Ligase Catalytic Subunit	The first rate-limiting enzyme of glutathione synthesis
	Gstm3	GSTM3 glutathione S-transferase mu 3	Mediates uptake and detoxification of both endogenous compounds and xenobiotics
	Gstm5	Glutathione S-transferase Mu 5	Conjugation of reduced glutathione to exogenous/endogenous hydrophobic electrophiles
	Sod1	Superoxide Dismutase 1	Eliminates radicals that are toxic to biological systems
Lipid Catabolism	Acot2	Acyl-CoA Thioesterase 2	Regulation of lipid metabolism/intracellular levels of free fatty acids
	Acox1	Peroxisomal acyl-coenzyme A oxidase 1	Catalyzes the desaturation of acyl-coas to 2-trans-enoyl-coas
	Cpt1b	Carnitine palmitoyltransferase 1b	Rate-controlling enzyme of fatty acid beta-oxidation
	Cyp4a10	Cytochrome p450 4a10	Arachidonic acid metabolic process
	Cyp4a12a	Cytochrome p450 4a12a	Metabolism of fatty acids and oxylipins
	Cyp4a14	Cytochrome p450 4a14	Oxidation of medium chain fatty acids
	Ehhadh	Enoyl-CoA Hydratase And 3-Hydroxyacyl CoA Dehydrogenase	Enzyme in fatty acid beta-oxidation pathway

## Data Availability

Publicly available datasets were analyzed in this study. This data can be found here: https://www.ebi.ac.uk/pride/archive (accessed on 27 April 2024).

## Supplementary Materials

The following supporting information can be downloaded at: https://www.mdpi.com/article/10.3390/toxics12050348/s1, Figure S1: Proteomic sample preparation workflow; Figure S2: PFOA, PFOS, PFHxS, and PFAS Mix altered protein expression in offspring liver; Figure S3: Individual and shared proteins within treatment comparisons—focusing on diet and PFAS-based effects.
