# Supplementary material for: Exposure to a PFOA, PFOS and PFHxS Mixture during Gestation and Lactation Alters the Liver Proteome in Offspring of CD-1 Mice
Source: Toxics. 2024 May 9;12(5):348. doi: 10.3390/toxics12050348 (PMC11126053; doi:10.3390/toxics12050348)
Supplement: Supplementary file 1 [file toxics-12-00348-s001.zip › toxics-2885924-supplementary.pdf]

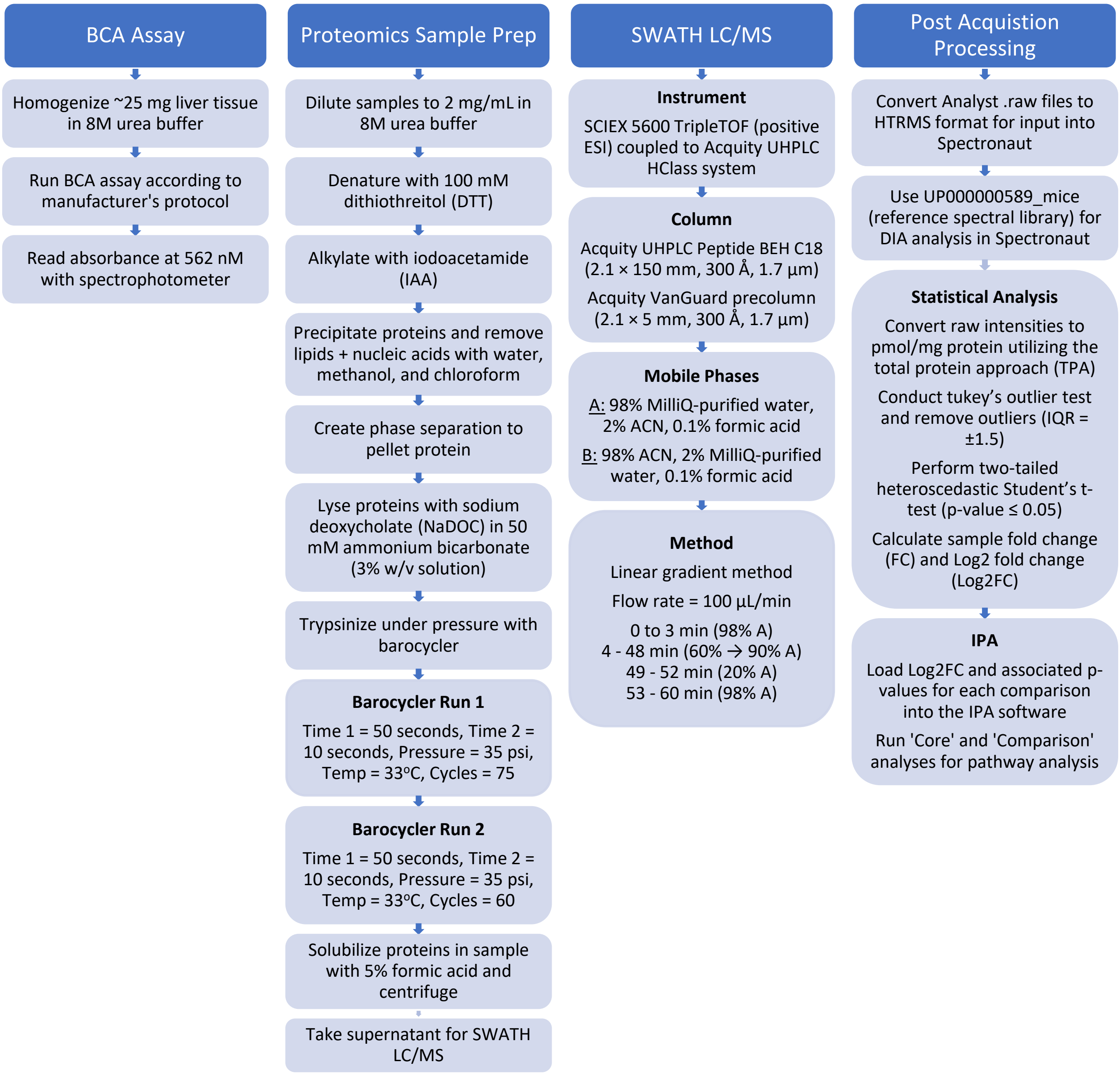

Figure S1. Proteomic sample preparation workflow. A simplified procedural workflow for proteomic sample preparation is visualized to help follow detailed methodology.

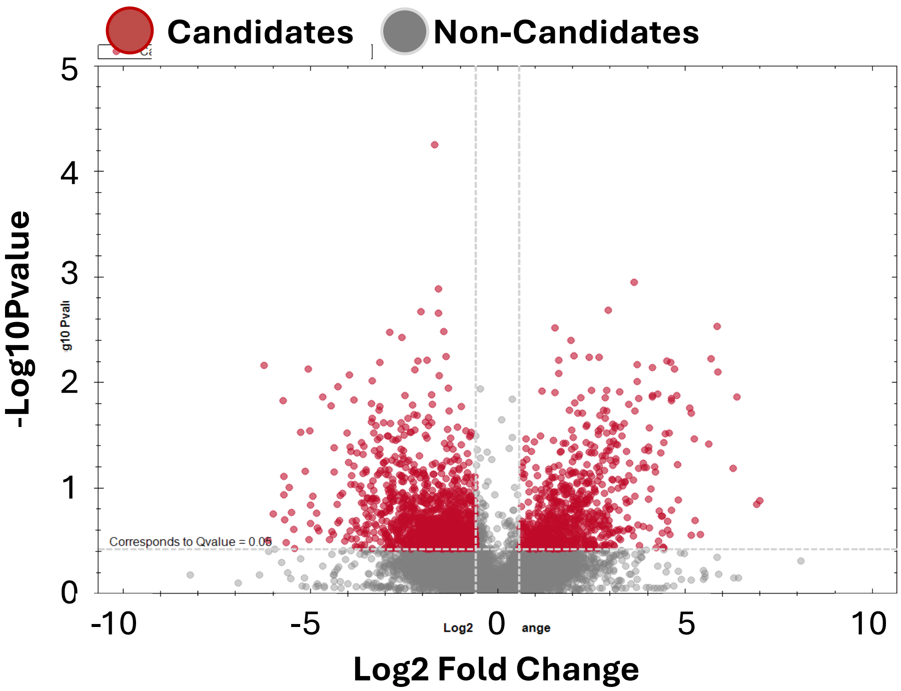

Figure S2. PFOA, PFOS, PFHxS, and PFAS Mix altered protein expression in offspring liver. There were 1,818 significantly altered proteins (p-val <0.05). 152 proteins were had increased expression and 123 had decreased expression.

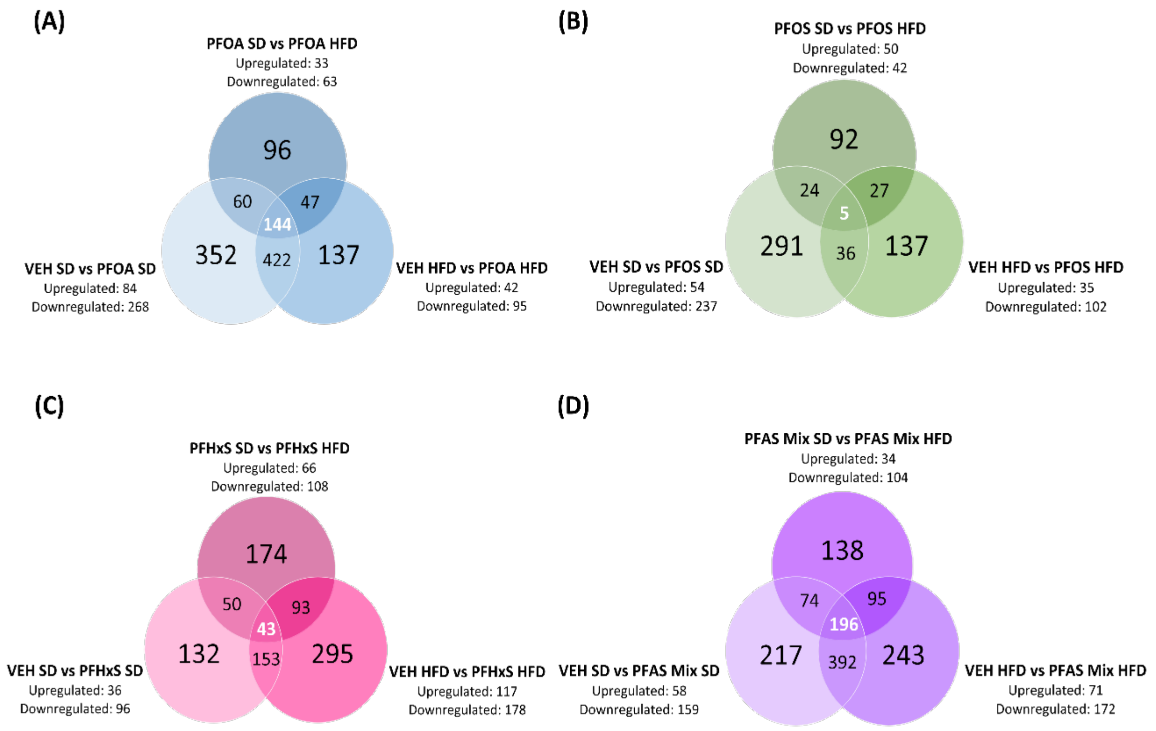

Figure S3. Individual and shared proteins within treatment comparisons - focusing on diet and PFAS-based effects. The Venn diagrams were created by Log2 transforming the treatment comparison fold changes of PND21 pups, and then further filtering out all insignificant Log2FC values ($p < 0.05$). Each Venn diagram aims to elucidate both exclusive and commonly expressed significant proteins between comparisons. There is a focus on investigating both diet and structure-based effects between PFAS treatment* verus VEH (both SD and HFD) and PFAS treatment* HFD versus PFAS treatment* (A) PFOA, (B) PFOS, (C) PFHxS, and (D) PFAS Mixture.

*PFAS Treatments include: PFOA, PFOS, PFHxS, and PFAS Mixture
